# The distinct phenotype of primary adipocytes and adipocytes derived from stem cells of white adipose tissue as assessed by Raman and fluorescence imaging

**DOI:** 10.1007/s00018-022-04391-2

**Published:** 2022-06-25

**Authors:** Ewa Stanek, Marta Z. Pacia, Agnieszka Kaczor, Krzysztof Czamara

**Affiliations:** 1 Jagiellonian Centre of Experimental Therapeutics (JCET), Jagiellonian University, 14 Bobrzynskiego Str., 30-348 Kraków, Poland; 2 Faculty of Chemistry, Jagiellonian University, 2 Gronostajowa Str., 30-387 Kraków, Poland

**Keywords:** Adipocytes, Adipose tissue, Stem cells, Raman microscopy, Fluorescence, Immunostaining, Adipogenesis

## Abstract

Spectroscopy-based analysis of chemical composition of cells is a tool still scarcely used in biological sciences, although it provides unique information about the cell identity accessible in vivo and in situ. Through time-lapse spectroscopic monitoring of adipogenesis in brown and white adipose tissue-derived stem cells we have demonstrated that considerable chemical and functional changes occur along with cells differentiation and maturation, yet yielding mature adipocytes with a similar chemical composition, independent of the cellular origin (white or brown adipose tissue). However, in essence, these stem cell-derived adipocytes have a markedly different chemical composition compared to mature primary adipocytes. The consequences of this different chemical (and, hence, functional) identity have great importance in the context of selecting a suitable methodology for adipogenesis studies, particularly in obesity-related research.

## Introduction

Growing interest in adipose tissue (AT) biology is correlated with dangerously expanding obesity-oriented health problems. Over 30 years of development studies on adipocytes proved that alterations within a certain stage of their differentiation affect its proper function and are linked to a multitude of disorders that are part of what is known as metabolic syndrome considered as a major cardiovascular risk factor [1].

AT is a multifunctional organ that provides whole-body energy regulation, thermogenesis, as well as endocrine and immunological support related to the ability of remodeling under the influence of pathophysiological conditions [2]. AT, based on its numerous characteristics, morphological diversity, and different anatomical locations, is a highly heterogeneous tissue that can be divided into brown, beige, pink, and white types [3]. AT, in addition to mature adipocytes, consists of the so-called stromal vascular fraction (SVF), a heterogeneous mesenchymal population of cells that include, among others, endothelial cells, erythrocytes, stem and progenitor cells, lymphocytes, and macrophages. SVF contains a subpopulation of elongated adherent cells that in the cell culture give the origin to multipotent ones termed adipose-derived stem cells (ASCs) [4]. This mesenchymal lineage of fibroblast-like cells has a remarkable ability to convert into progenitors characteristic to the musculoskeletal system, i.e., adipocytes, for tissue engineering applications. In particular, ASCs are essential to initiate the tightly orchestrated process of adipogenesis [5]. Disruptive ASCs maturation is intertwined with the systemic AT inflammation observed in obesogenic states but is also paralleled with the physiological downregulation of lipid metabolism, cellular composition, and changes in synthesized adipokines [6]. Therefore, there is an evident demand for a more in-depth look at the population of SVF cells, especially in the context of cellular impairment.

Despite many contemporary techniques, from highly advanced electron microscopy to more straightforward biochemical methods, Raman spectroscopy is increasingly used in the biomedical field, for example, in clinical trials related to regenerative cell pathways, diagnostics and disease development [7–10]. Taking into account the rich presence of nonpolar acyl chains in the structure of the lipids, a robust signal originating from their typical bands can be identified in the tissue, in many cases as a manifestation of dysfunction [11, 12]. The lipid-rich AT is a particularly convenient tissue for studying using Raman-based techniques. Therefore, recently, this methodology was applied to investigate changes in perivascular adipose tissue (PVAT) due to the development of lifestyle diseases including diabetes [13], insulin resistance [14], and cardiovascular diseases such as atherosclerosis [15]. Raman spectroscopy was also used in ex vivo studies of brown (BAT) and white adipose tissue (WAT) to distinguish vibrational differences arising from their function and morphology in healthy [16] and obese [17, 18] states. These studies demonstrated that Raman microscopy, due to its low invasiveness and elimination of lengthy sample preparation processes, is a promising tool in AT lipidomics and, prospectively, will contribute further insight into malfunctioned metabolic processes in AT.

Extensive research on AT has shown that lipids have promising diagnostic applicability, especially in the context of obesity, and are linked to their prognostic parameters. Accordingly, it is a need to determine which model of lipid metabolism gives the closest representation of the truth of homeostatic regulation [19]. Therefore, lipid profiling of various commercially available murine cell lines was performed. Results indicated differences between adipocytes derived from mesenchymal stem cells and defined preadipocyte lines [20]. To date, the maturation of ASCs has been examined using conventional Raman microscopy [21, 22] and its variants such as coherent anti-Stokes Raman scattering imaging (CARS) [23, 24] or stimulated Raman spectroscopy (SRS) [10, 25].

Unique capabilities of Raman microscopy to investigate the chemical composition and component distribution may provide new information regarding adipogenesis in the cell cultures, in particular about the course of this process in cells of different origins. Therefore, this work aimed to monitor the development of cell cultures of SVF cells isolated from WAT and BAT depots, that is, epididymal and interscapular adipose tissues (eWAT and iBAT, respectively) to verify the changes in the phenotype of both types of AT during adipogenesis, also referring them to the phenotype of primary adipocytes. The focus was put on the chemical composition of de novo lipid droplets (LDs) formation at the selected time points of adipogenesis. For this purpose, cells were monitored during successive stages of maturation. Based on characteristic bands of the collected Raman spectra, the degree of lipid unsaturation was calculated and compared within the studied groups of cultured adipocytes derived from SVF cells from eWAT and iBAT and primary adipocytes extracted from these depots. Our results suggest, *inter alia*, that adipocytes considerably differ in the chemical composition of lipids whether they are derived from cell culture or have been freshly isolated from the tissue. This is critical in the context of selecting a methodology for experiments conducted in biological studies, especially in terms of obesity-related research.

## Methods

### Cell isolation and culture

To establish a high purity and homogenous culture of premature adipocytes, several steps were utterly followed. AT depots of eWAT and iBAT were isolated from five 10 weeks-old C57Bl/6J male mice (Medical University of Bialystok, Bialystok, Poland) without cutting into the surrounding muscles, glands, and other organs to avoid cross-contamination. Next, all fragments of AT were mixed, minced and underwent a reported isolation procedure [26] with additional modifications. The tissue digestion was performed for 1 h in the enzymatic solution composed of 3.5 mg/mL collagenase D (Roche Holding AG, Basel, Switzerland), 2% bovine serum albumin (BSA, Sigma), and 150 µM CaCl_2_ dissolved in PBS at 37 °C in a water bath with gentle shaking every 10 min until AT became cloudy followed by filtration through a 100 μm nylon cell strainer. After that, SVF cells were separated by centrifugation (280 g, 3 min, RT) from floating primary adipocytes which subsequently placed between CaF_2_ microscope slide and the coverslip were ready for the upcoming Raman measurements. The SVF cell pellet was suspended in red blood cell lysis buffer (155 mM NH_4_Cl, 12 mM NaHCO_3_, 0.1 mM EDTA) for three minutes at room temperature, centrifuged and immediately resuspended at the required concentration in a fresh medium (MI, DMEM: F12 (Gibco Life Technologies) that contained 20% fetal bovine serum (FBS, Gibco Life Technologies) and 50 µg/mL gentamycin (Sigma), and placed directly on CaF_2_ slides for further spectroscopic imaging. After 24 h attached cells were washed with a fresh portion of the MI medium and by the third day about 90% confluence was obtained. Induction of differentiation was performed by switching MI for medium II (MII, i.e., DMEM:F12 supplemented with 8% FBS, 8 µg/mL biotin (Sigma), 50 µg/mL gentamycin (Sigma), 1.15 µg/mL insulin (Sigma), 80 µg/mL 3-isobutyl-1-methylxanthine (IBMX, Sigma), 1.5 µg/mL of troglitazone (Sigma) and 0.4 µg/mL of dexamethasone (Sigma) for 4 days. Subsequently, MII was changed to the maintenance medium (MIII i.e. DMEM:F12 containing 8% FBS, 8 µg/mL biotin, 50 µg/mL gentamycin) for another 7 days. For Raman imaging, cells were fixed with 2.5% glutaraldehyde solution for 10 min, washed twice, kept in PBS, and stored at 4 °C up to the measurements. The analysis was performed on cells after each culture medium representing fibroblast-like cells, preadipocytes, and SVF-derived adipocytes, and also cells on days 2, 4, and day 7 of adipocytes maturation to monitor the morphological and chemical changes during cell growth.

All experimental procedures involving animals were conducted according to the Guidelines for Animal Care and Treatment of the European Communities and the Guide for the Care and Use of Laboratory Animals published by the US National Institutes of Health (NIH Publication No. 85–23, revised 1996).

### Raman microscopy

Raman imaging was performed using a WITec confocal Raman microscope (WITec alpha300, Ulm, Germany) equipped with a 532 nm laser, a UHTS 300 spectrograph (600 grooves·mm^−1^ grating), and a CCD detector (DU401A-BV-352, Andor, UK). Raman spectra were acquired with a 0.5 s exposure time per spectrum using the maximum laser power (*ca*. 30 mW) at the sample. As samples were measured in the buffer, potential damages due to the laser were eliminated. For each group, 5–8 cells were measured. 3D Raman profiling was conducted by measuring the selected cell area changing the focal distance in a 1 µm step in the *z* axis that receives 8–9 layers per cell. The distribution images at different depths present the relative intensity of nuclei, endoplasmic reticulum, and LDs. Raman spectra of cultured cells were measured on CaF_2_ slides with the application of the 60 × water immersion objective (NA = 1.0, Zeiss Fluor, Germany) and for the primary adipocytes using a 20 × air objective (NA = 0.45, Nikon CFI S Plan Fluor ELWD, Japan) due to the different sample preparation technique.

### Immunohistochemical stainings: UCP-1 and perilipin-1 expression

Before staining, cells were preincubated with 5% normal donkey serum (Jackson Immuno Research), 2% dry milk, and 1% TritonX100 in PBS to reduce nonspecific binding. Then, cells were immunostained using rabbit anti-mouse anti-UCP-1 (Abcam; 1:100) and goat anti-mouse anti-Perilipin-1 (Abcam; 1:100) primary antibodies overnight. For fluorescent detection of UCP-1 and perilipin-1, the protocol was performed in three steps: 1./Immunostaining of UCP-1: after rinsing in PBS, the secondary biotinylated donkey anti-rabbit Ig (JacksonImmuno; 2:600) was applied. After 30 min cells were rinsed in PBS and incubated with Cy3-conjugated streptavidin (JacksonImmuno; 2:600) for 30 min to visualize the primary antibody binding sites. 2./Blocking of unspecified binding sites: after rinsing in PBS, 1 mg/mL biotin (Sigma Aldrich) in PBS was used. After 15 min, cells were rinsed in PBS and incubated with streptavidin (Sigma Aldrich; 2:600) for 15 min. 3./Immunostaining of perilipin-1: after rinsing in PBS, the secondary biotinylated donkey anti-goat Ig (JacksonImmuno; 2:600) was applied. After 30 min cells were rinsed in PBS and incubated with Alexa Fluor 488-conjugated streptavidin (JacksonImmuno; 2:600) for 30 min and washed in distilled water.

Images were acquired using an AxioCam HRm digital monochromatic camera and an AxioObserver.D1 inverted fluorescent microscope (Carl Zeiss). The UCP-1 or perilipin-1 fluorescence was quantified by counting the area of the intensity of UCP-1 or perilipin-1 signal vs. the area of whole cells using ImageJ software (National Institutes of Health, USA).

### Data analysis and processing

Preprocessing was done using the WITec Project Plus software, obtained Raman spectra were baseline corrected using an autopolynomial of degree 3 and then submitted to the cosmic ray removal technique. Then, cluster analysis (KMCA i.e., the *k*-means method using the Manhattan distance, WITec Project Plus) was used to separate the class rich in lipids and the average spectra reflecting the composition of the lipid droplets. Spectra of lipids were normalized using vector normalization in the 3100–2800 cm^−1^ or 1800–400 cm^−1^ spectral ranges using the OPUS 7.2 program. Moreover, the integral intensities of the bands at 1744, 1656, 1442 cm^−1^ were calculated in 1770–1722, 1701–1630, and 1516–1392 cm^−1^ spectral ranges, respectively. The ratio of the integral intensities of bands located at 1656/1442 cm^−1^ was used to determine the degree of lipid unsaturation. All data were compared in the Origin Pro 9.1 program using the ANOVA variance analysis with the Tukey post hoc test to characterize the differences in the chemical distribution in all pairwise comparisons for each of the studied groups. If the *p* parameter was 0.05, differences were identified as statistically significant.

## Results

### Time-dependent alterations in the chemical composition of maturing adipocytes in monitoring adipogenesis

**Fig. 1:**
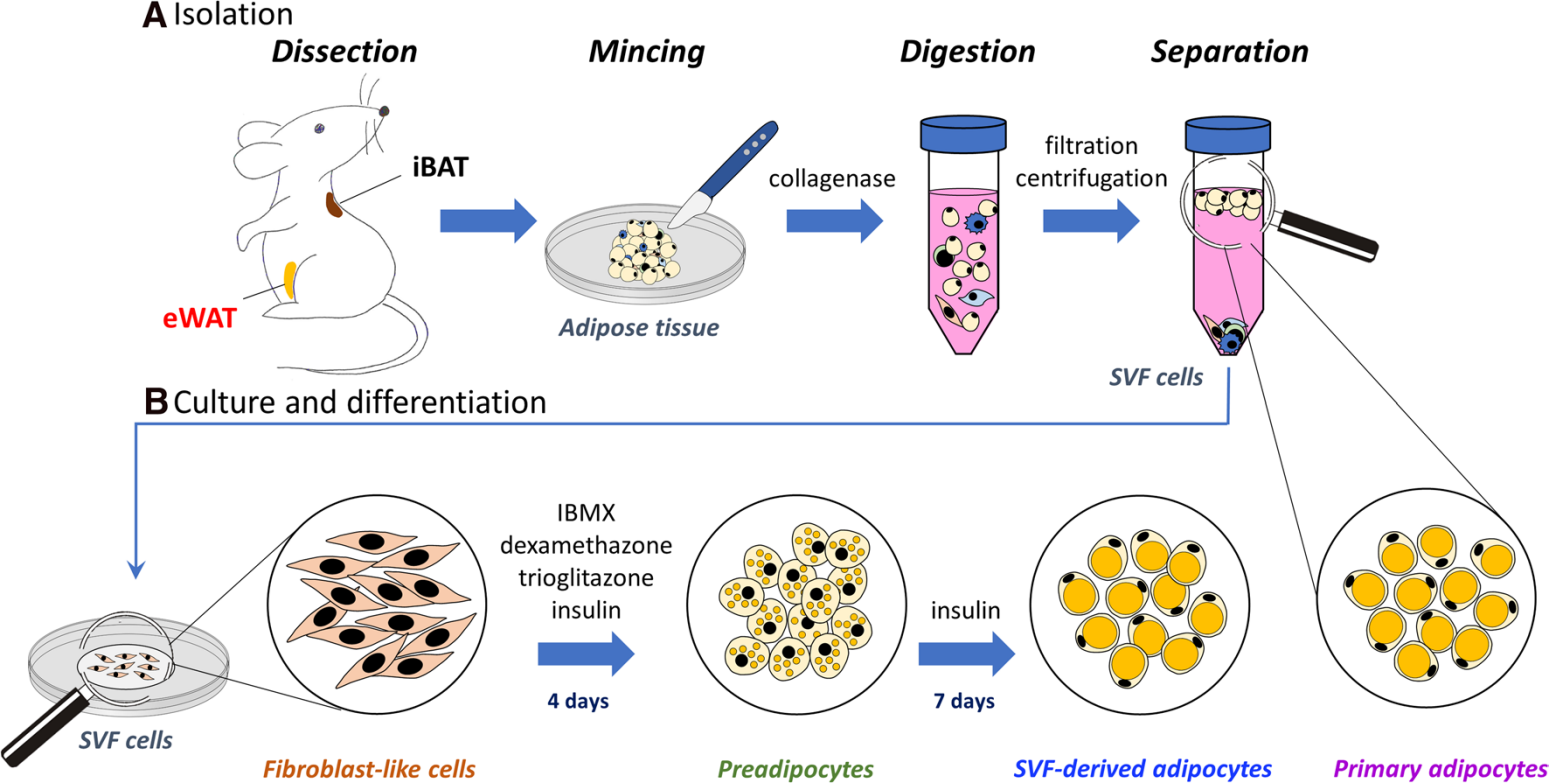
The flow scheme showing the isolation of primary adipocytes and procedures necessary to obtain culture of SVF-derived adipocytes. Samples of the interscapular (iBAT) and epididymal white adipose tissue (eWAT) were isolated from C57Bl/6J mice at the age of 10 weeks, then subjected to the procedures outlined above. Cell culture procedure was performed according to the literature [26]

The in vitro research performed on adipocytes can be conducted on freshly isolated primary adipocytes or SVF-originating preadipocytes after maturation. The isolation protocol was the same for both cell types in the early steps until the separation step. The scheme of procedures necessary for the preparation of cells for Raman microscopy is presented in Fig. 1.

**Fig. 2:**
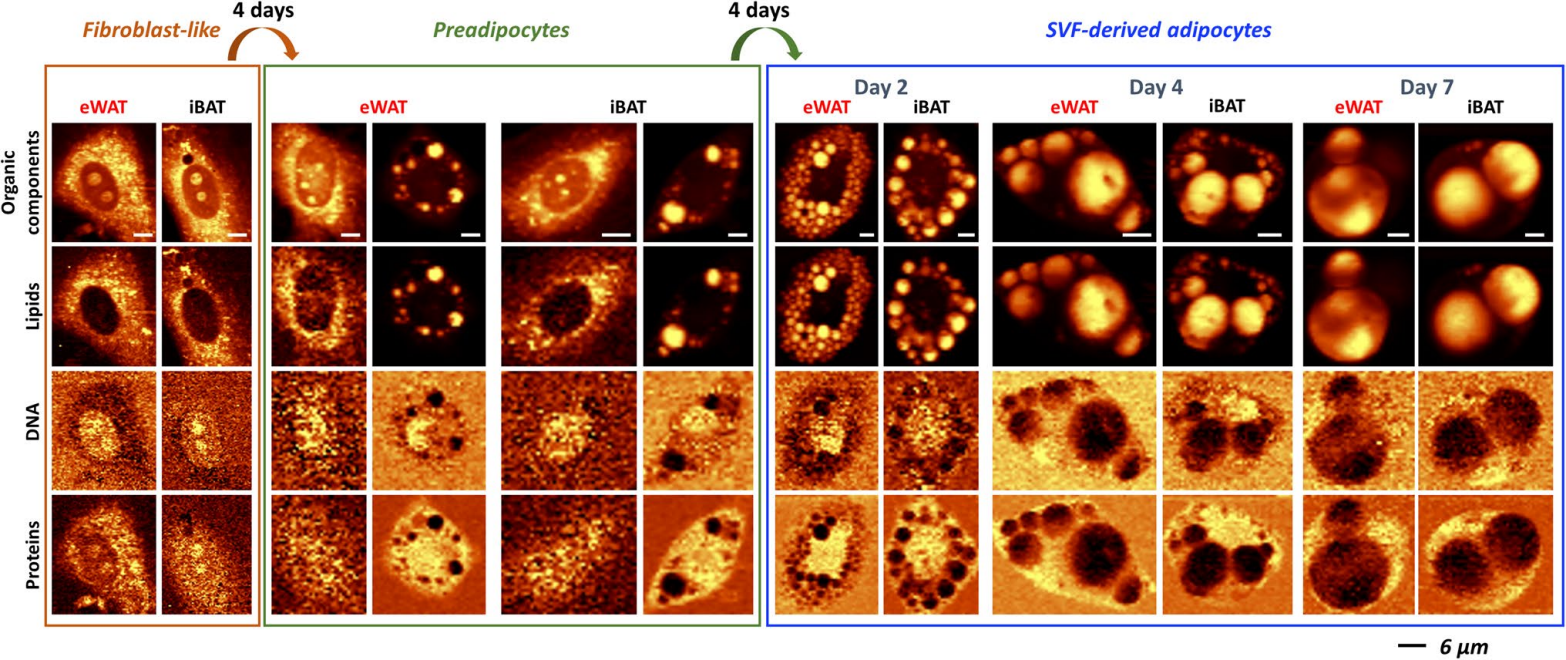
Distinct Raman distribution images of SVF-derived cells in the early and late stages of adipogenesis. Raman images were obtained by integration of the bands in the following spectral regions: 3030–2800 cm^−1^ (organic matter), 2900−2830 cm^−1^ (lipids), 805−770 cm^−1^ (DNA), and 1025–995 cm^−1^ (proteins)

The selection of eWAT and iBAT was dictated by their different phenotypes and adhesive properties. To investigate the maturation of adipocytes, SVF cells from eWAT and iBAT were introduced into the cell culture. After each media set and appropriate fixation, Raman microscopy was performed. To monitor the changes in the subcellular architecture and composition, two-dimensional Raman images were obtained by the integration of the selected marker bands (Fig. 2). Raman distribution images, based on the bands arising from the C–H stretching vibrations, corresponding to all organic matter, show alterations in the cell morphology. Moreover, Raman distribution images of nuclei were obtained based on ring breathing modes in the DNA nitrogen bases as well as C′_5_–O–P–O–C′_3_ phosphodiester bands [27]. A special focus was laid on lipids, which are mainly seen in the endoplasmic reticulum and inside LDs (integration of the C–H stretching vibrations in the spectral range 2900–2830 cm^−1^). In addition, Raman images of proteins were shown to visualize their distribution in the cell cytoplasm (integration of the phenylalanine breathing mode).

**Fig. 3:**
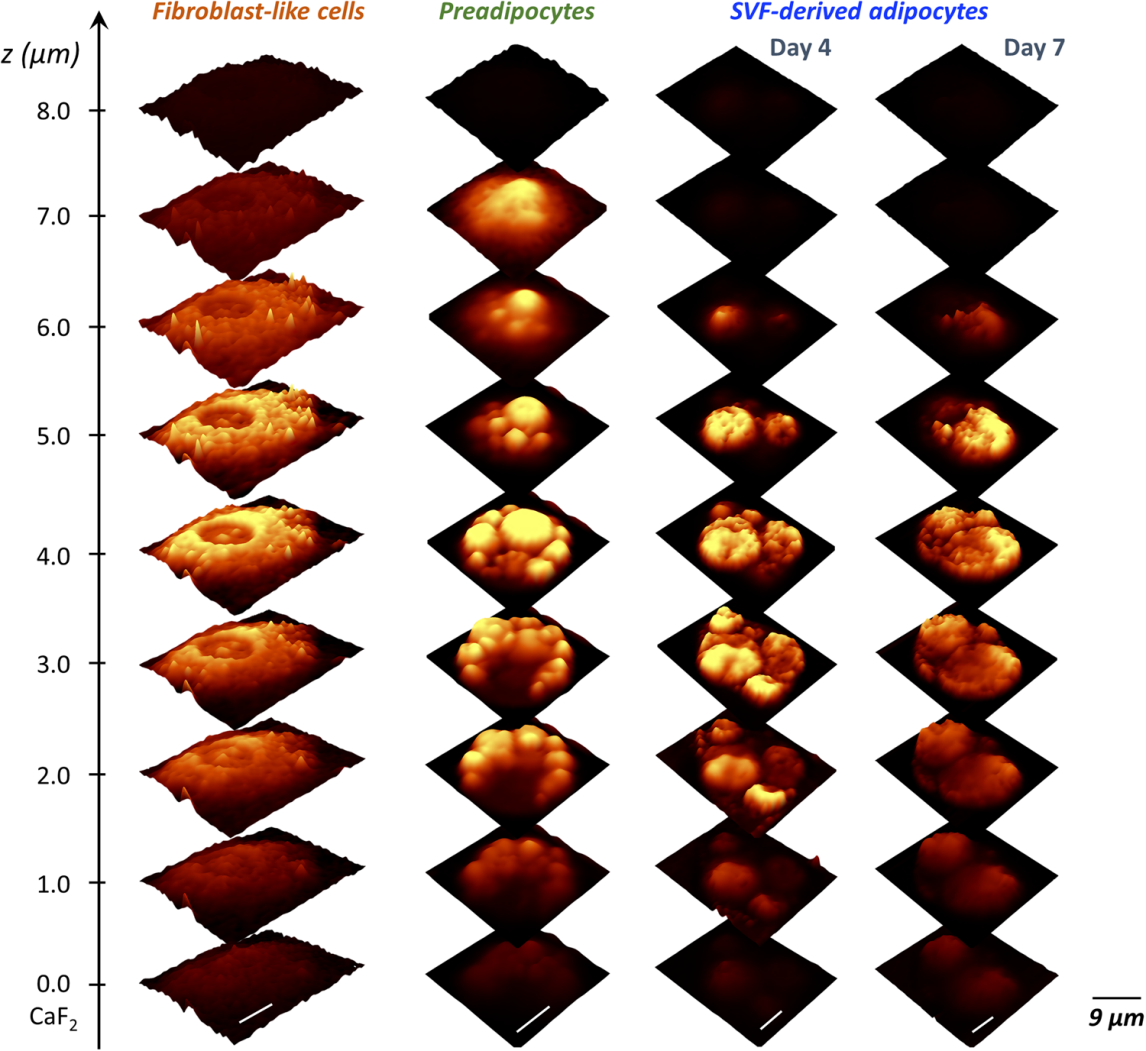
Three-dimensional Raman profiling of representative SVF-derived cells (eWAT type) during various stages of adipogenesis. Images were obtained by integration of the bands in the 3030–2800 cm^−1^ spectral range for cells after application of each cell culture medium. The signal was normalized between layers for images per group separately

To initiate and carry on adipogenesis, SVF cells were cultured in specific cell culture media that ultimately affected adipogenic cell precursors and put on the maturation pathway [26]. The first medium (MI) promotes growth and assimilation to the new environment, the second (MII) induces differentiation and contains ingredients that harness cells to enter the preadipocyte state, and the third (MIII) upholds the overall maintenance of the cell culture where almost matured adipocytes start more visibly increase the volume of LDs. After the MI medium, only one fraction of cells was observed for eWAT and iBAT (Fig. 2) i.e., cells that maintained the morphology typical for fibroblasts with a clearly distinguishable nucleus and the endoplasmic reticulum, which is the presumable source of LDs forming at further stages of development. After the MII medium, a substantial fraction of cells changed their morphology, shrunk taking to a more oval shape, and generated clearly outlined LDs assuming the morphology of preadipocytes. Additionally, fibroblast-like cells with smaller LDs were observed in this fraction. The parallel co-existence of the two subpopulations is due to the lack of synchronicity in the cell cycle of the original cell population and the heterogeneous response of individual cells to pro-differentiation signals. After MIII, most cells were mature with massive LDs found within cells. Raman images of the lipid distribution show a progression in LDs size up to the point that LDs eventually fuse forming a uniform lipid area. After 7 days, a multitude of cells with large LDs was observed with thinned confluence, which is a consequence of decreased adherence of mature adipocytes and their detachment from the surface. Both 2D (Fig. 2) and 3D (Fig. 3) Raman images show clear differences in the morphology of the cells and the chemical composition of the lipids during the maturation process.

### Spectral changes during adipogenesis are due to changes of the lipid unsaturation degree and level of triacylglycerols

**Fig. 4:**
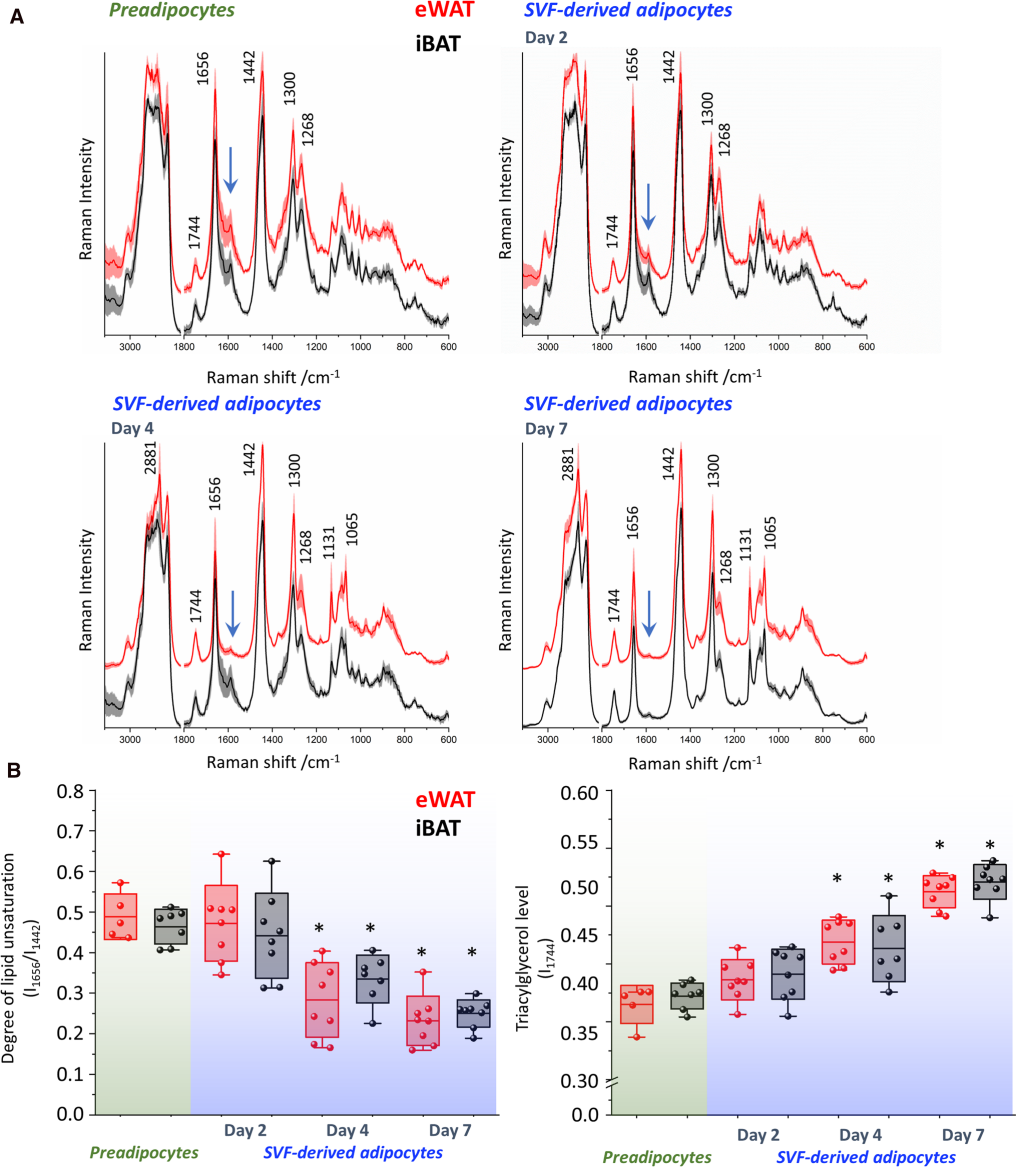
Changes of Raman spectra and chemical composition of SVF-derived cells in the early and late stages of adipogenesis. Average spectra (**A**) normalized in the 3100–2800/1800–400 cm^−1^ spectral ranges and presented with the standard deviation for all data sets. The degree of lipid unsaturation (I_1656_/I_1442_) and triacylglycerol level (I_1744_) were calculated for each AT depot (**B**). Values shown in box plots: mean (horizontal line), SD (box), minimal and maximal values (whiskers). Statistical significance *p < *0.05* with respect to the MII group (preadipocytes)

In the next step of the work, a detailed analysis of the lipid composition in cells was performed. For this purpose, cluster analysis, enabling the extraction of the information separately from the LDs class. The presented average spectra (Fig. 4A) show the composition of LDs in the subpopulation of preadipocytes (after using MII) and the population of adipocytes after using MIII for each investigated day. The average spectra of cells in the same group (preadipocytes or adipocytes on days 2, 4, and 7) for eWAT and iBAT are practically indistinguishable. The only detectable exception (marked with a blue arrow) is found for iBAT in the range 1580–1590 cm^−1^ and at 752 cm^−1^ that is related to hemeproteins, typical for BAT (presumably reflecting increased mitochondrial activity). These alterations are particularly evident in adipocytes day 2 and day 4 groups.

Regardless of the type of studied cells, the Raman profile of LDs resembles a typical spectrum of unsaturated triacylglycerols as evidenced by the specific 1744 cm^−1^ band, the carbonyl stretching vibration of ester moieties [28]. Based on the ratio of the calculated integral intensities of the bands at 1656/1442 cm^−1^ corresponding to C=C stretching mode and CH_2_ scissoring and twisting modes, respectively, the degree of lipid unsaturation can be quantified (Fig. 4B). As the processes involved in adipogenesis continue, the spectral profile changes dramatically after the following days of maturation. The most distinct changes observed from day 4 are the significant decrease in the intensity of bands 1656 and 1268 cm^−1^ vs. 1442 and 1300 cm^−1^ and the appearance of signals at 1131, 1065, 892 cm^−1^ and intense peak at 2881 cm^−1^ in high wavenumber region, indicating the change in lipid composition towards more saturated lipids. These observations correlate with the increasing content of triacylglycerols that accumulate in LDs (Fig. 4B). In general, during adipogenesis, for both AT types, the increase in triacylglycerol levels in cells, accompanied by a decrease in their lipid unsaturation degree, were detected.

### Morphological but not chemical or functional differences occurred between SVF-derived adipocytes of WAT and BAT

**Fig. 5:**
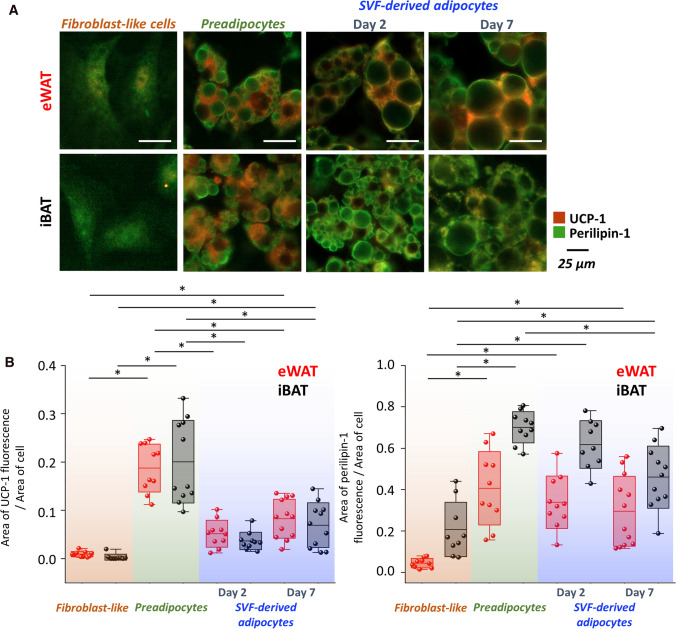
Distinct changes of UCP-1 and perilipin-1 expression in preadipocytes and SVF-derived adipocytes. Representative images (**A**) of cells from simultaneously stained for UCP-1 (red fluorescence) and perilipin-1 (green fluorescence). The quantitative analysis of ratios of positively stained UCP-1 or perilipin-1 areas to the total area of the cell (**B**). Values shown in box plots: mean (horizontal line), SD (box), minimal and maximal values (whiskers). Statistical significance *p < *0.05*

The lack of differences in the chemical composition of LDs in BAT and WAT indicated that the differentiation and maturation of SVF cells are not dependent on their origin. To check whether the functional aspect was maintained, the UCP-1 and perilipin-1 immunostainings were performed (Fig. 5), visualizing UCP-1 or the LDs’ membrane, respectively.

Figure 5 shows that during differentiation and maturation, SVF cells undergo significant morphological evolution from elongated and flattened cells to roundish ones. Moreover, mature adipocytes originating from eWAT, especially on day 8 MIII, possess clearly visible large LDs surrounded by positively stained spots for perilipin-1 in comparison to iBAT where LDs and cells are smaller. The analysis of the UCP-1 positive area (red pixels) reveals the elevated mitochondrial activity during the differentiation process which subsequently decreases with adipocytes maturation regardless of AT type. Parallel changes are observed in perilipin-1 activity. For preadipocytes, the area of perilipin-1 positive area (green pixels) is the highest indicating numerous newly formed LDs within cells. Due to the fusion of LDs during maturation, the perilipin-1 intensity decreases, however, there is no significant difference between AT types. Overall, the examined WAT and BAT SVF-derived adipocytes, although have somehow different morphology and size of LDs, exhibit similar UCP-1 and perilipin-1 expression. The immunostainings confirm the results obtained by Raman imaging.

### Different chemical composition of primary versus SVF-derived adipocytes

To evaluate the effects of the selected protocol for differentiating SVF cells, two types of adipocytes extracted from eWAT and iBAT, i.e. freshly isolated primary and those obtained under culture conditions in vitro from selected adipose tissue depots were critically compared*.*

**Fig. 6:**
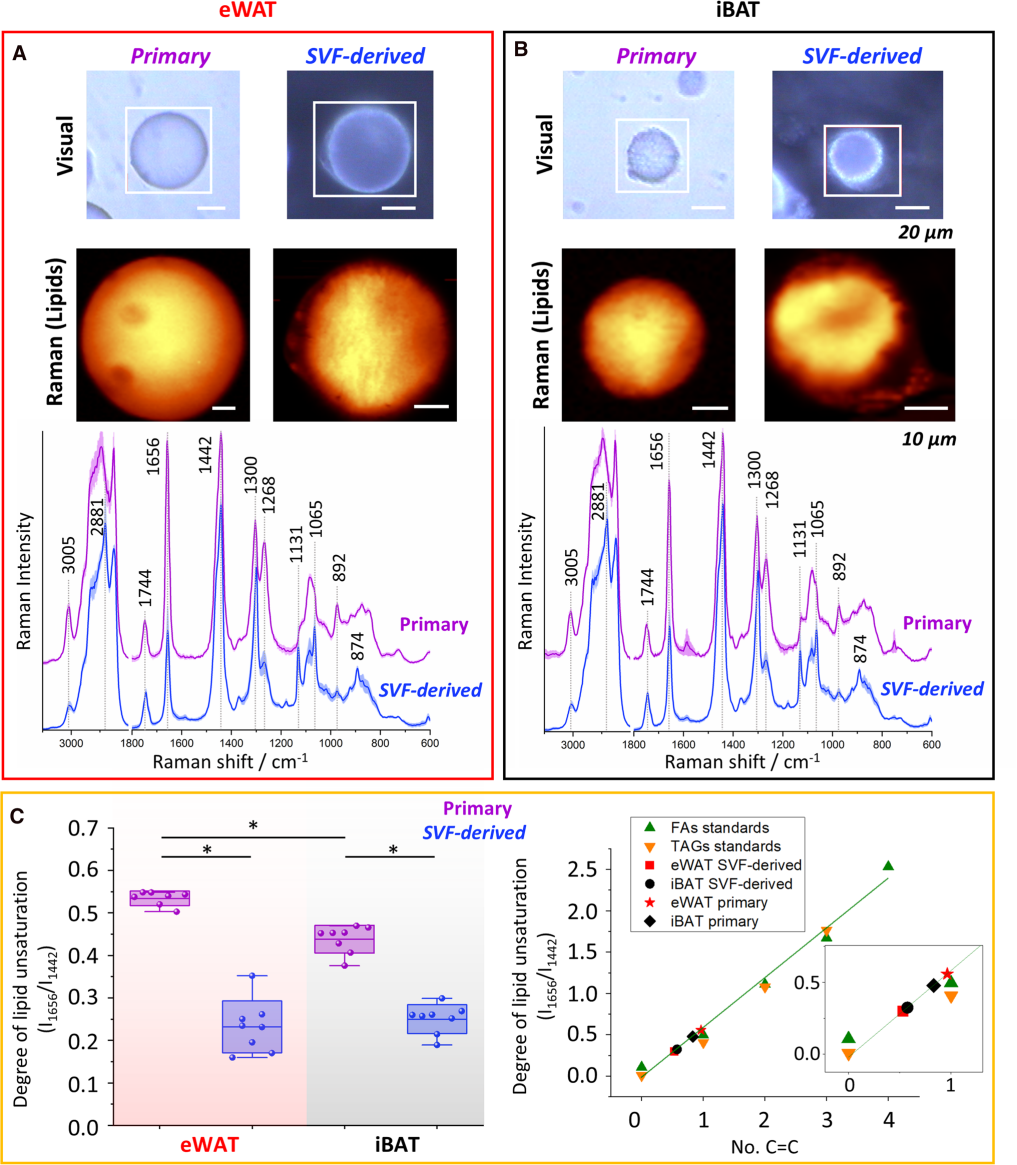
Differences in the chemical composition of primary and SVF-derived mature adipocytes. Microscopic and Raman images of primary and SVF-derived mature adipocytes (day 7 after MIII) for eWAT and iBAT with averaged Raman spectra (**A**, **B**). Degree of lipid unsaturation (based on I_1656_/I_1442_) determined for each AT depot and presented in box plots (the number of C=C double bonds was based on the calibration curve, **C**). Values shown in box plots: mean (horizontal line), SD (box), minimal and maximal values (whiskers). Statistical significance *p < *0.05*

Despite the quite high morphological similarity observed with both microscopic and Raman images, the spectral profile of primary adipocytes is considerably different from the cultured ones (Fig. 6A, B). Major differences are seen for the following bands: 3005, 1656, 1442, 1300, 1131, 1065, and 892 cm^−1^ corresponding to vibrations associated with the lipid unsaturation and hydrocarbon chains. Simultaneously with these findings, the differences between primary white and brown adipocytes are also prominent, as previously observed in studies of AT [29]. Estimation of the degree of lipid unsaturation indicates that lipids of primary adipocytes present a higher unsaturation degree than those of SVF-derived mature cells whose Raman profile resembles the spectrum of glyceryl palmitate [28]. To define the chemical composition of LDs, the results were compared to standards for fatty acids and triacylglycerols based on the number of double bonds they contained. On that basis, after calculating the degree of lipid unsaturation, the values were fitted to a calibration curve [30] (Fig. 6C). The SVF-derived mature adipocytes from WAT and BAT matched more closely to each other with a value of *ca*. 0.5 for C=C bonds confirming their more saturated Raman profile. These results also highlighted subtle differences between primary WAT and BAT adipocytes. Although lipids in white adipocytes are more unsaturated, both types contain higher amounts of monounsaturated fatty acids than SVF-derived adipocytes and maintain the differences presented for the tissues [15].

## Discussion

Undoubtedly, the last couple of decades of fat cell investigations shed light on its metabolic functions and the key pathways that govern it. The pursuit of a better understanding physiologically induced processes in AT has resulted in the implementation of various cell line models for in vitro testing. As an assessment of biological models, they are widely available and commonly used to screen their response under a multitude of chemical agents including pharmacological factors [31]. However, despite their advantages, a variety of problems are frequently arising since up to 50% of adipocyte cell lines are being mislabeled, which in the end leads to delusive scientific conclusions [32]. Furthermore, because of their simplified nature, they should not be taken as a realistic instrument to portray one-to-one physiology. One reason is the absence of cells with which they interact in the organism, secondly, the culture media used do not usually contain all nutrients needed. In lipidomic analyses, the availability of essential fatty acids supplied exogenously from the diet is particularly vital. Furthermore, the transport of fatty acids to AT is mediated by several lipoproteins circulating in the serum [19], which could be lacking in standard supplemental protocols. To examine these intricacies, more methods should be included. One of them is high-resolution Raman microscopy which finds useful application as a non-invasive approach and a supporting technique for biomolecular research in a wide range of lifestyle diseases [33, 34]. Thanks to inelastic interactions of photons with matter it provides a multitude of information about the chemical composition of the sample. Hence, we used it to seek an association between shifts in lipidome matter and the state of cellular integrity during structural conversions in evolving AT.

By application of Raman microscopy, we were able to not only observe changes in the morphology of the obtained cells but also successfully assign spectra specific to all cell classes. The images of lipids distribution (Fig. 2) due to the integration of the 2900–2830 cm^−1^ band showed that the endoplasmic reticulum and the LDs formed within it play a major role in the subsequent phases of fat cell development. This agrees with the current view on LDs biogenesis [35] and other spectroscopic studies like Raman mapping [21] or coherent anti-Stokes Raman scattering microscopy (CARS) [36] in the context of adipogenesis. The 3D Raman images also provided insight into physical parameters (Fig. 3) such as height, number of LDs, their arrangement with each other, and the cell nucleus. Considering that WAT adipocytes have a small, peripherally located nucleus in their structure [37], we are ultimately unable to see it at the end of adipogenesis due to the overshadowing signal of lipids. Overall, on the succeeding days of maturation, fibroblast-like cells became more lipid-rich, which is related to the uptake of fatty acids from the environment and their conversion into neutral lipids accumulated in LDs [38].

The next complication in AT research is the substantial heterogeneity of AT depots, therefore currently the large-scale testing of lipidomics and differences in lipid composition of existing WAT and BAT-like lines [20] with sex-specific distinction is undertaken [39]. A comparison of differentiated adipocytes from precursor cells from various AT depots was also performed [40]. Due to its functions, the lipid profile varies depending on the type of tissue from which the adipocyte is derived. WAT stores energy in the form of triacylglycerols, BAT dissipates this energy in thermogenesis, hence they are mainly studied for different lipid classes responsible for diverse activities. These classes are divided into several groups and their content depends on tissue metabolism. The major variation between the two types of AT lies in the number of shorter acyl chain triacylglycerols with less saturated characteristics, of which there are far more in WAT. Another difference is the longer chain of unsaturated fatty acids in BAT, which is associated with the role of elongases in the thermogenesis process. Brown adipocytes also possess lower levels of free fatty acids, that are required for beta-oxidation and activation of the UCP-1 receptor [20]. Moreover, BAT exhibits more saturated triacylglycerols, which is potentially driven by the fact that saturated fatty acids produce more ATP than polyunsaturated ones [41].

Interestingly, we were unable to observe significant spectral differences in SVF-derived adipocytes from WAT and BAT (Fig. 4A), which may be due to the protocol used to differentiate SVF cells [26]. For instance, in a study comparing 3T3-L1 preadipocytes, ear mesenchymal progenitor cells, and brown adipose-derived BAT-C1 cells, the 3T3-L1 line was supplemented with 100 × higher concentrations of insulin than BAT, which may have been relevant to the ultimate chemical composition of emerged adipocytes [20]. Beyond that, studies are showing that impaired glucose uptake shifts BAT to store energy, which is called “whitening”, therefore, in the end, this tissue gains partially WAT characteristics [42]. Since in the work of Kwiecien et al. [26] the protocol was applied only to WAT, this effect may also be visible on the BAT, hence the high similarity between them.

The fluorescence imaging shed more light on SVF-derived adipocyte functionality (Fig. 5). The analysis of UCP-1 and perilipin-1 expressions have shown that the differentiation, enhanced by dexamethasone and IBMX, which were used to stimulate the glucocorticoid receptor and the PPAR-γ expression, and intracellular cAMP and protein kinase pathways [43, 44], respectively, led to the increased UCP-1 activity in preadipocytes that diminish during maturation of adipocytes. Our results are consistent with the well-known fact that the differentiation process involves elevated mitochondrial biogenesis and up-regulates characteristic mitochondrial proteins, that is, UCP-1 [45]. Additionally, Kladnická et al. [46] reported for human adipose-derived mesenchymal stem cells that the mitochondrial network increased the most in the first 10 days of differentiation before the significant increase in lipid accumulation. Moreover, the low UCP-1 expression in mature adipocytes and the perilipin-1 immunostaining show the accelerated lipid accumulation induced by insulin [47], indicating the rather white-like characteristic of SVF-derived cells for both types of AT. Parallelly, the evolution of the number and size of LDs from small and numerous to a few large occurred.

Lipid chemical composition changes upon adipogenesis. Raman spectra clearly show the increase of intensity of characteristic bands arising from the hydrocarbon chain i.e. 1131, 1065, 1300, and 2881 cm^−1^ indicating the increase of lipid saturation (Fig. 4A). Additionally, ratiometric analysis of the degree of lipid unsaturation demonstrated that the decrease in lipids accumulated in cells over successive days is correlated with an increase in the level of triacylglycerols (Fig. 4B), which has not matched the results of primary adipocytes freshly isolated from both depots (Fig. 6). These results highlight two important aspects. In WAT promoting conditions, determined by applied protocol, the behavior of SVF-derived cells and their change of phenotype to typical for WAT (low expression of UCP-1), is quite expected, however, for BAT indicated their ability for phenotype conversion. Surprisingly, both SVF-derived adipocytes arising from WAT and BAT possess similar but different than primary WAT adipocytes lipid chemical composition manifested by the lower value of the degree of lipid unsaturation. Assuming that primary adipocytes reflect the actual state present in the tissue in vivo, we can conjecture that the outputs generated in culture cannot be compared with each other one-to-one in future tests, especially bearing in mind the effect of different factors in media buffers. This fact has already been raised for other cell types of vasculature i.e. for endothelium [48], highlighting the differences between primary and differentiated cells. For AT, as we know, the presence of unsaturated fatty acids in LDs in a quite significant amount is necessary to help mammalian cells in diminishing the toxic aftermath of saturated fat by promoting triacylglycerols production [49]. Given the above, the priority should be to customize the culture conditions to the tissue type in terms of the components that are needed along with the maturation under the conditions found in the organism. In this manner, the final set of cells should carry a chemical profile similar to that observed in vivo. This will limit the false interpretation of the studied outcomes.

## Conclusions

AT provides a uniquely abundant and accessible source of stem cells that can be adjusted to specific scientific purposes, i.e. studies of adipogenesis. Using Raman and fluorescence imaging we proved that the chemical composition of lipids and functionality of mature SVF-derived adipocytes do not depend on the source of cells and the WAT or BAT origin blurs for adipocytes obtained by differentiation and maturation of stem cells dependently on the applied protocol. Moreover, we demonstrated that in WAT-like evoking conditions SVF-derived mature adipocytes from eWAT differ markedly from the primary adipocytes in chemical composition while showing a similar morphology. This outcome may be of high importance for in vitro studies of adipocytes’ lipid metabolism and may have an impact on potential applications in tissue engineering in i.e. microphysiological systems.

## Data Availability

All data generated or analyzed during this study are included in this published article.

## Abbreviations

ASCs: Adipose-derived stem cells
AT: Adipose tissue
BAT: Brown adipose tissue
CARS: Coherent anti-Stokes Raman scattering
eWAT: Epididymal white adipose tissue
iBAT: Interscapular brown adipose tissue
LDs: Lipid droplets
PVAT: Perivascular adipose tissue
SRS: Stimulated Raman spectroscopy
SVF: Stromal vascular fraction
UCP-1: Uncoupling protein 1
WAT: White adipose tissue

## References

[1] Wang QA, Scherer PE, Gupta RK (2014). Improved methodologies for the study of adipose biology: insights gained and opportunities ahead. J Lipid Res.

[2] Choe SS, Huh JY, Hwang IJ (2016). Adipose tissue remodeling: its role in energy metabolism and metabolic disorders. Front Endocrinol.

[3] Giordano A, Smorlesi A, Frontini A (2014). White, brown and pink adipocytes: the extraordinary plasticity of the adipose organ. Eur J Endocrinol.

[4] Corrêa, Heyn, Magalhaes (2019). The impact of the adipose organ plasticity on inflammation and cancer progression. Cells.

[5] Han S, Sun HM, Hwang K-C, Kim S-W (2015). Adipose-derived stromal vascular fraction cells: update on clinical utility and efficacy. Crit Rev Eukaryot Gene Expr.

[6] Mancuso P, Bouchard B (2019). The impact of aging on adipose function and adipokine synthesis. Front Endocrinol.

[7] Ember KJI, Hoeve MA, McAughtrie SL (2017). Raman spectroscopy and regenerative medicine: a review. npj Regen Med.

[8] Pacia MZ, Czamara K, Zebala M (2018). Rapid diagnostics of liver steatosis by Raman spectroscopy: via fiber optic probe: a pilot study. Analyst.

[9] Marzec KM, Rygula A, Gasior-Glogowska M (2015). Vascular diseases investigated ex vivo by using Raman, FT-IR and complementary methods. Pharmacol Reports.

[10] Ferrara MA, Filograna A, Ranjan R (2019). Three-dimensional label-free imaging throughout adipocyte differentiation by stimulated Raman microscopy. PLoS ONE.

[11] Pacia MZ, Sternak M, Mateuszuk L (2020). Heterogeneity of chemical composition of lipid droplets in endothelial inflammation and apoptosis. Biochim Biophys Acta - Mol Cell Res.

[12] Czamara K, Stojak M, Pacia MZ (2021). Lipid droplets formation represents an integral component of endothelial inflammation induced by lps. Cells.

[13] Proniewski B, Bar A, Kieronska-Rudek A (2021). Systemic administration of insulin receptor antagonist results in endothelial and perivascular adipose tissue dysfunction in mice. Cells.

[14] Bar A, Kieronska-Rudek A, Proniewski B (2020). In vivo magnetic resonance imaging-based detection of heterogeneous endothelial response in thoracic and abdominal aorta to short-term high-fat diet ascribed to differences in perivascular adipose tissue in mice. J Am Heart Assoc.

[15] Czamara K, Majka Z, Sternak M (2020). Distinct chemical changes in abdominal but not in thoracic aorta upon atherosclerosis studied using fiber optic raman spectroscopy. Int J Mol Sci.

[16] Giarola M, Guella G, Mariotto G (2008). Vibrational and structural investigations on adipose tissues. Philos Mag.

[17] Donjuán-Loredo G, Espinosa-Tanguma R, León-Bejarano F (2021). Raman spectroscopy for adipose tissue assessment in rat models of obesity and type 1 diabetes. Appl Spectrosc.

[18] Haka AS, Sue E, Zhang C (2016). Noninvasive detection of inflammatory changes in white adipose tissue by label-free Raman spectroscopy. Anal Chem.

[19] Lamaziere A, Farabos D, Wolf C, Quinn PJ (2013). The deficit of lipid in cultured cells contrasted with clinical lipidomics. Mol Nutr Food Res.

[20] Liaw L, Prudovsky I, Koza RA (2016). Lipid profiling of in vitro cell models of adipogenic differentiation: relationships with mouse adipose tissues. J Cell Biochem.

[21] Gomathy SS, Stylianou C, Phang IY et al (2010) Raman mapping glucose metabolism during adipogenesis from human mesenchymal stem cells. In: 2010 Photonics global conference. pp 1–5. 10.1109/PGC.2010.5705978

[22] Mitchell A, Ashton L, Yang XB (2015). Detection of early stage changes associated with adipogenesis using Raman spectroscopy under aseptic conditions. Cytom Part A.

[23] Downes A, Mouras R, Bagnaninchi P, Elfick A (2011). Raman spectroscopy and CARS microscopy of stem cells and their derivatives. J Raman Spectrosc.

[24] Di Napoli C, Pope I, Masia F (2014). Hyperspectral and differential CARS microscopy for quantitative chemical imaging in human adipocytes. Biomed Opt Express.

[25] den Broeder MJ, Moester MJB, Kamstra JH (2017). Altered adipogenesis in zebrafish larvae following high fat diet and chemical exposure is visualised by stimulated raman scattering microscopy. Int J Mol Sci.

[26] Kwiecien K, Brzoza P, Bak M (2020). The methylation status of the chemerin promoter region located from − 252 to + 258 bp regulates constitutive but not acute-phase cytokine-inducible chemerin expression levels. Sci Rep.

[27] Movasaghi Z, Rehman S, Rehman IU (2007). Raman spectroscopy of biological tissues. Appl Spectrosc Rev.

[28] Czamara K, Majzner K, Pacia MZ (2015). Raman spectroscopy of lipids: a review. J Raman Spectrosc.

[29] Czamara K, Majka Z, Fus A (2018). Raman spectroscopy as a novel tool for fast characterization of the chemical composition of perivascular adipose tissue. Analyst.

[30] Pacia MZ, Majzner K, Czamara K (2020). (2020) Estimation of the content of lipids composing endothelial lipid droplets based on Raman imaging. Biochim Biophys Acta - Mol Cell Biol Lipids.

[31] Lafontan M (2012). Historical perspectives in fat cell biology: the fat cell as a model for the investigation of hormonal and metabolic pathways. Am J Physiol - Cell Physiol.

[32] Babic Z, Capes-Davis A, Martone ME (2019). Incidences of problematic cell lines are lower in papers that use RRIDs to identify cell lines. Elife.

[33] Majka Z, Czamara K, Janus J (2022). (2022) Prominent hypertrophy of perivascular adipocytes due to short-term high fat diet. Biochim Biophys Acta - Mol Basis Dis.

[34] Maase M, Rygula A, Pacia MZ (2019). Combined Raman- and AFM-based detection of biochemical and nanomechanical features of endothelial dysfunction in aorta isolated from ApoE/LDLR−/− mice. Nanomedicine Nanotechnology, Biol Med.

[35] Olzmann JA, Carvalho P (2019). Dynamics and functions of lipid droplets. Nat Rev Mol Cell Biol.

[36] Nan X, Cheng JX, Xie XS (2003). Vibrational imaging of lipid droplets in live fibroblast cells with coherent anti-Stokes Raman scattering microscopy. J Lipid Res.

[37] Park A (2014). Distinction of white, beige and brown adipocytes derived from mesenchymal stem cells. World J Stem Cells.

[38] Onal G, Kutlu O, Gozuacik D, Dokmeci Emre S (2017). Lipid droplets in health and disease. Lipids Health Dis.

[39] Hoene M, Li J, Häring HU (2014). The lipid profile of brown adipose tissue is sex-specific in mice. Biochim Biophys Acta - Mol Cell Biol Lipids.

[40] Schweizer S, Liebisch G, Oeckl J (2019). The lipidome of primary murine white, brite, and brown adipocytes—Impact of betaadrenergic stimulation. PLoS Biol.

[41] Lapid K, Graff JM (2017). Form(ul)ation of adipocytes by lipids. Adipocyte.

[42] Lapa C, Arias-Loza P, Hayakawa N (2017). Whitening and impaired glucose utilization of brown adipose tissue in a rat model of type 2 diabetes mellitus. Sci Rep.

[43] Rosen ED, Spiegelman BM (2000). Molecular regulation of adipogenesis. Annu Rev Cell Dev Biol.

[44] Scott MA, Nguyen VT, Levi B, James AW (2011). Current methods of adipogenic differentiation of mesenchymal stem cells. Stem Cells Dev.

[45] Kusminski CM, Scherer PE (2012). Mitochondrial dysfunction in white adipose tissue. Trends Endocrinol Metab.

[46] Kladnická I, Čedíková M, Kripnerová M (2019). Mitochondrial respiration of adipocytes differentiating from human mesenchymal stem cells derived from adipose tissue. Physiol Res.

[47] Cignarelli A, Genchi VA, Perrini S (2019). Insulin and insulin receptors in adipose tissue development. Int J Mol Sci.

[48] Kennedy CC, Brown EE, Abutaleb NO, Truskey GA (2021). Development and application of endothelial cells derived from pluripotent stem cells in microphysiological systems models. Front Cardiovasc Med.

[49] Fujimoto T, Parton RG (2011). Not just fat: the structure and function of the lipid droplet. Cold Spring Harb Perspect Biol.

